# Caught in vicious circles: a perspective on dynamic feed-forward loops driving oxidative stress in schizophrenia

**DOI:** 10.1038/s41380-021-01374-w

**Published:** 2021-11-10

**Authors:** Michel Cuenod, Pascal Steullet, Jan-Harry Cabungcal, Daniella Dwir, Ines Khadimallah, Paul Klauser, Philippe Conus, Kim Q. Do

**Affiliations:** 1 Center for Psychiatric Neuroscience, Department of Psychiatry, Lausanne University Hospital (CHUV), Prilly, Lausanne, Switzerland; 2 Service of Child and Adolescent Psychiatry, Department of Psychiatry, Lausanne University Hospital, Prilly, Lausanne, Switzerland; 3 Service of General Psychiatry, Department of Psychiatry, Lausanne University Hospital, Prilly, Lausanne, Switzerland

**Keywords:** Diagnostic markers, Schizophrenia, Prognostic markers, Physiology, Neuroscience

## Abstract

A growing body of evidence has emerged demonstrating a pathological link between oxidative stress and schizophrenia. This evidence identifies oxidative stress as a convergence point or “central hub” for schizophrenia genetic and environmental risk factors. Here we review the existing experimental and translational research pinpointing the complex dynamics of oxidative stress mechanisms and their modulation in relation to schizophrenia pathophysiology. We focus on evidence supporting the crucial role of either redox dysregulation, N-methyl-D-aspartate receptor hypofunction, neuroinflammation or mitochondria bioenergetics dysfunction, initiating “vicious circles” centered on oxidative stress during neurodevelopment. These processes would amplify one another in positive feed-forward loops, leading to persistent impairments of the maturation and function of local parvalbumin-GABAergic neurons microcircuits and myelinated fibers of long-range macrocircuitry. This is at the basis of neural circuit synchronization impairments and cognitive, emotional, social and sensory deficits characteristic of schizophrenia. Potential therapeutic approaches that aim at breaking these different vicious circles represent promising strategies for timely and safe interventions. In order to improve early detection and increase the signal-to-noise ratio for adjunctive trials of antioxidant, anti-inflammatory and NMDAR modulator drugs, a reverse translation of validated circuitry approach is needed. The above presented processes allow to identify mechanism based biomarkers guiding stratification of homogenous patients groups and target engagement required for successful clinical trials, paving the way towards precision medicine in psychiatry.

Schizophrenia research faces many challenges due to the disease complexity and heterogeneity at various levels, from genetic, pathophysiology to clinical phenomenology and stages. Early detection and intervention [1] requires mechanism-based reliable biomarkers that capture circuitry dysfunction, allowing better patient stratification, disease progression monitoring and treatment. To this goal, it is essential that experimental research on animal models is coupled with translational clinical observations [2]. This review reports attempts to uncover mechanisms underlying schizophrenia pathophysiology at molecular, circuitry, system and cognitive levels, and identify novel preventive and therapeutic measures.

A longstanding pathophysiological approach to schizophrenia emphasizes the role of abnormal neurodevelopment in relation to long-term alterations of neural circuits that lead to the emergence of disease symptoms [3, 4]. Prominent theories associated with the pathogenesis of schizophrenia include dopamine, glutamate/NMDA [5, 6], neuroimmune/neuroinflammatory [7], mitochondrial hypotheses [8], and excessive microglia-mediated synaptic pruning [9, 10], while deficits in gamma-aminobutyric acid (GABA) system and myelination are well documented [11, 12]. Oxidative stress (OxS) has emerged as a “central hub” in schizophrenia pathophysiology given the converging evidence from environmental and genetic studies. They link this physiological process to cardinal pathological features of the disease including alterations in both parvalbumin-expressing GABAergic neurons (PV neurons) (microcircuits) and myelinated macrocircuits [13, 14].

Here, we propose the hypothesis that a dysfunction during development in either NMDAR-mediated signaling, neuroimmune regulation, mitochondria function could initiate “vicious circles” centered on redox dysregulation/OxS, leading to persistent anomalies of PV neurons and oligodendrocytes and ultimately to neural synchronization, cognitive, emotional, social and sensory deficits characteristic of schizophrenia (Fig. 1). The concept of OxS-driven PV neuron impairment is supported by our recent study assessing prefrontal PV interneurons in a range of animal models carrying genetic and/or environmental risk factors of schizophrenia affecting glutamatergic, dopaminergic, immune and redox signaling [15]. The present paper expands this by reviewing the experimental and clinical evidence pinpointing the complex dynamics of OxS mechanisms and their modulation in relation to schizophrenia pathophysiology.

**Fig. 1:**
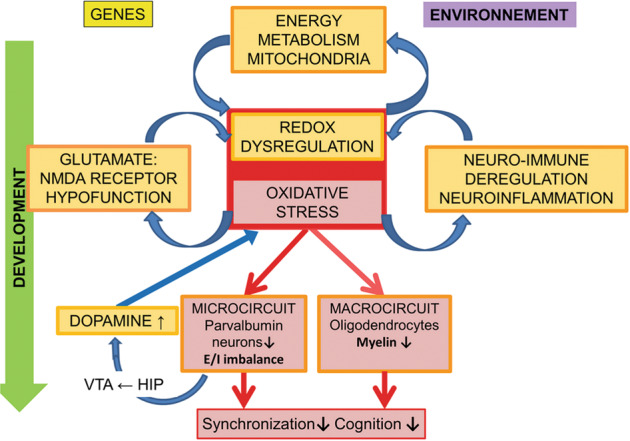
Schematic representation of the concept proposed in this paper, showing the reciprocal interaction between mitochondria, NMDAR, neuro-immune system, dopamine on one hand and the complex redox regulation/oxidative stress (OxS) on the other. Irrespectively of which is the primary affected system via genetic or environmental factors, this will trigger during development subsequent vicious circles of OxS that can feed on one another and drive durably parvalbumin (PV) neurons and myelin impairments that culminate in the neural synchronization and cognitive deficits characteristic of schizophrenia.

## Interactions between redox dysregulation and oxidative stress

The redox balance between reactive oxygen species (ROS) and antioxidant systems is critical in the brain, which displays a high oxidative metabolism, as compared to other organs. Maintenance of redox homeostasis typically involves the delicate regulation of ROS by redox systems, with evidence that the glutathione (GSH/GSSG), thioredoxin and cysteine/cystine redox systems are differentially modulated under dynamic and non-equilibrium redox conditions [16]. These systems control redox signaling (i.e.Nrf2 redox-signaling pathway) and redox-sensing within cells. Notably, redox-sensing cysteine residues (i.e., thiol switches) provide an orthogonal control system to modulate activity of cellular and physiological mechanisms [17]. Severe and chronic unbalance between ROS and antioxidant systems would lead to oxidative damage on proteins, lipids and DNA with drastic irreversible effects. However, a slight redox dysregulation would lead to reversible oxidation of the thiol switch on redox-sensitive proteins, leading to their functional modifications. This can alter receptor-(NMDAR) and kinase-mediated signaling (Fyn kinase), metalloprotease (MMP9) activity, thus affecting neurotransmission, and cellular proliferation, differentiation, maturation in case of transient redox dysregulation during development [18]. Below, we will focus on the critical role of GSH, without discarding that OxS could also result from dysregulation of other antioxidant systems (thioredoxin [19], peroxiredoxin, sulforedoxin [20]).

Since our first observations on alterations of GSH metabolism in cerebrospinal fluid of schizophrenic patients in the nineties [21, 22], evidence that redox dysregulation plays a major role in psychosis has gained prominence. Data accumulated over the last decades point to increased OxS (increased lipid and protein oxidation) and alterations in antioxidant defence systems (vitamin C and E, catalase and superoxide dismutase) in blood, plasma cerebrospinal fluid and post-mortem samples of schizophrenia patients [18, 23–27]. Various genes directly involved in antioxidant systems have been associated with risk for schizophrenia. They include the catalytic (GCLC) [28] and modulatory (GCLM) [29] subunits of glutamate-cysteine ligase (rate-limiting synthesizing enzyme of GSH), glutathione-S-transferase [30, 31], superoxide dismutase-1 [32], nitric oxide synthase [33, 34]. Genetic variations within some of these genes exert strong functional effects on phenotypes. Thus, carriers of the high-risk GCLC genotype (GAG trinucleotide repeat polymorphism) exhibit lower GSH levels in fibroblasts when challenged with OxS [28], and lower prefrontal GSH levels compared with GCLC low-risk genotypes [13, 28, 35, 36]. Additional research has revealed epigenetic alterations in GSH genes in at-risk individuals who later converted to psychosis [37].

Using in-vivo magnetic resonance spectroscopy, we first reported decreased GSH levels in prefrontal cortex (PFC) of drug naive patients [22]. In line with these findings, recent work has revealed lower levels of GSH in anterior cingulate cortex and thalamus of schizophrenia patients [38, 39]. Although some studies with small numbers of subjects did not observe such decrease [40, 41], recent meta-analyses support a GSH deficit in anterior cingulate cortex [42, 43]. Interestingly, in early psychosis patients, low prefrontal GSH levels were associated with high-risk GCLC genotypes, highlighting GCLC polymorphisms should be considered in pathology studies of cerebral GSH [36]. In first episode psychosis, higher GSH prefrontal levels were associated with favorable prognosis [44]. Reduced levels of GSH in post-mortem brains of schizophrenia patients have also been reported [45–47]. In this context, several groups have assessed peripheral blood GSH levels as a window to understand what occurs in the brain [36, 48], although it remains unclear whether the reported changes in peripheral GSH levels are a reflection of the brain ones (see also caveats of plasma GSH levels analysis in [18] supplement). Recently, we have demonstrated a negative correlation between high GSH peroxidase (GPx) activity in red blood cells and low brain GSH levels in male early psychosis patients [36]. As such, blood GPx activity may reflect central oxidative status, although these findings require validation in larger cohorts.

## Vulnerability of PV neurons to redox dysregulation

### PV neurons in schizophrenia

One of the most consistent pathological findings in schizophrenia are anomalies in PV neurons and their associated extracellular matrix, the perineuronal net (PNN) [49]. Primarily reported in PV interneurons of the hippocampus (reduced number of PV-immunoreactive neurons) [50, 51] and dorsolateral PFC (i.e. abnormal PNN, decreased PV and GAD67 expression) [52], anomalies have been also observed in other cortical and subcortical regions [53–58], and cerebellum [59]. Abnormal function of these diverse PV neurons affects high-frequency (gamma) neuronal synchronization within brain regions and cortico-thalamic network dynamic [60, 61], impacting sensory processing, attention, working memory, learning, social behavior, fear processing, motor coordination and learning, and contributing to hyperdopaminergia related to positive symptoms [62–69]. As estrogen interacts extensively with PV neurons [70], the drop in this hormone at menopause could also possibly contribute via altered function of these neurons to the increased incidence of late-onset schizophrenia in women. Thus, PV neuron dysfunction appears to be a core of schizophrenia pathophysiology. All these PV neurons are GABAergic inhibitory neurons that can generate action potentials at very high frequency. Therefore, they require considerable energy to sustain intense neuronal activity as during high-frequency synchronization. In this perspective, hemodynamic signals correlate positively with high gamma oscillations [71]. Optimal functional performance of mitochondria [72] is essential to support such strong demand for adenosine triphosphate (ATP) produced by oxidative phosphorylation [73]. This metabolic process can generate ROS through electron leak, thus making PV neurons particularly vulnerable to redox imbalance. One cannot exclude that other micro-circuit-related GABAergic neurons, including the somatostatin ones [74] as well as pyramidal cells [75] could be directly or indirectly affected.

### Preclinical evidence

Cumulating evidence indicates that PV neurons are indeed vulnerable to redox dysregulation—stemming from a compromised antioxidant system or ROS overproduction. Although most preclinical studies have focused on hippocampal and prefrontal PV interneurons, PV neurons are also affected by redox dysregulation in other regions, including thalamus reticular nucleus [56, 76], amygdala [77], globus pallidus [77], inferior colliculus [78]. Overall, PV neurons are impacted when antioxidant systems (e.g., GSH, selenoprotein P, catalase, superoxide dismutase) are compromised [78–80]. In a transgenic mouse expressing low GSH levels (Gclm KO), we found reduced number of prefrontal and hippocampal PV-immunoreactive interneurons together with diminished high-frequency neuronal synchronization [81, 82], reduced number of PV-immunoreactive neurons in thalamus reticular nucleus together with altered firing properties [56], thus demonstrating the functional consequence of a redox dysregulation. In Gclm KO mice and other models involving a weakened antioxidant capacity, the OxS is more prominent in PV neurons as compared to other types of GABAergic or principal neurons [78–80]. Notably, OxS precedes PV neuron deficits [82] and is accompanied by a weakening of the PNN enwrapping them [77, 81, 82]. These alterations are reversed by the antioxidant N-acetyl-cysteine (NAC), confirming the causal role of OxS. Other works found that superoxide overproduction by NADPH oxidase (NOX) has deleterious effects on PV neurons, with evidence that NOX inhibition prevents PV neuron impairment induced by either NMDAR antagonist [76] or social isolation [83].

Remarkably, we further showed that PV neuron deficit in anterior cingulate cortex is associated with OxS in a variety of animal models carrying genetic and/or environmental risks relevant to diverse etiological aspects of schizophrenia [15]. Specifically, OxS correlates negatively with the integrity of PV neurons and their PNN [15]. Overall redox dysregulation/OxS appear as a common pathological mechanism leading to PV neuron-associated network anomalies in schizophrenia (Fig.1).

### Developmental perspective

PV neurons are more susceptible to a redox dysregulation resulting in OxS during postnatal development rather than later in life [81, 84]. Indeed, a transient GSH deficit induced by L-buthionine-(S,R)-sulfoximine during early postnatal life causes long-term reduction of PV neuron density in anterior cingulate cortex [85–87]. Likewise, a permanent reduction of PV neuron density in anterior cingulate cortex of Gclm KO mice occurs following the administration of GBR-12909 (dopamine re-uptake inhibitor leading to excess extracellular dopamine level that generates ROS through its catabolism) during postnatal development, but not adulthood [81]. The vulnerability of immature PV neurons has been associated with the absence of yet fully mature PNN [81]. Indeed, PNN plays a key role in protecting PV neurons from OxS [81], but also in promoting their maturation. The maturation and integrity of PV neurons require incorporation of the non-cell autonomous homeobox protein Otx2 via its binding to the PNN [88, 89]. Thus, PV neurons during early postnatal development are less protected from OxS that also disrupts PNN formation, leading to long-term impairment of PV maturation and stabilization of synapses within their networks. The OxS-induced degradation of aggrecan-enriched PNN is mediated by metalloproteinases, including MMP9 [90, 91]. Of note, the mRNA expression pattern in PV neurons of schizophrenia patients indicate an immature state [92], including altered expression of genes regulating cell cycle and apoptosis [93]. Reduced expression of PV appears mediated in part by epigenetic mechanisms [94, 95].

The implication of redox dysregulation/OxS in the abnormal development of PV neurons has been further corroborated in neurodevelopmental animal models of schizophrenia that do not involve direct manipulation of the redox system. Adult rats with a neonatal ventral hippocampal lesion display OxS, reduced number of PV-immunoreactive interneurons, and weakened PNN in medial PFC which could be prevented through juvenile and adolescence treatment with NAC, or ebselen [84]. Likewise, early postnatal injection of ketamine leads in adulthood to OxS and decreased number of prefrontal PV-immunoreactive interneurons that is precluded by adolescent NAC treatment [80]. Finally, adult rats that have received the mitotoxin Methylazoxymethanol Acetate (MAM) during late gestation show deficits in hippocampal and prefrontal PV neurons, weakened PNN, and impaired neuronal synchronization alongside with OxS and decreased brain GSH levels [15, 96–98]. Moreover, OxS induced by a prenatal stress slows down the migration of inhibitory interneuron progenitors, a migration that can be accelerated by antioxidants [99]. Altogether, these indicate that OxS during development disrupts maturation and function of PV neuron-associated networks.

### Critical period of plasticity

Given that aperture and closure of the critical period of plasticity involves PV neuron maturation in conjunction with PNN and myelin formation—all of which are sensitive to disturbances in redox homeostasis — a redox dysregulation may disrupt critical periods during neurodevelopment. Thus, the neocortex of mice remains plastic beyond its typical critical period when redox dysregulation is restricted to PV neurons [100]. We therefore speculate that PV neuron-specific regulation of redox state may play a role in balancing plasticity and stability of cortical networks during development, relevant to distractibility, basic symptoms and disorders of the self-perception known to be central to the phenomenology of schizophrenia. Furthermore, the vulnerability to stresses is linked to the critical period [101] which is characterized by immature and not yet fully formed PNN unable to protect PV neurons from OxS-induced damage [81]. That is, mis-timed developmental trajectories of brain plasticity stemming from redox dysregulation may confer susceptibility to environmental stresses and risk for neurodevelopmental disorders such as schizophrenia [102].

## Reciprocal interactions between mitochondrial dysfunction and redox dysregulation/oxidative stress

Cumulating evidence exists linking mitochondrial dysfunction and oxidative phosphorylation generating ROS to schizophrenia [103–109]. In-vivo ^31^P-magnetic resonance spectroscopy revealed direct and compelling evidence for brain bioenergetics abnormality in schizophrenia patients [110]. This includes altered expression of mitochondria-related genes in prefrontal layer-III-PV neurons [111] and of the oxidative phosphorylation pathway resulting in OxS in interneurons derived from induced pluripotent stem cells [112]. Acute metabolic stress induced by environmental factors (infection and psychosocial stress) known to be associated with schizophrenia can trigger pervasive OxS in neurons [113], leading to mitochondrial dysfunction, which in turn, generates more ROS and neuronal damage. PV neurons have high mitochondrial content, due to the energy demand required to sustain their fast-spiking characteristics. This renders them particularly susceptible to OxS and mitochondrial damage [114].

### Preclinical evidence

Mice with a deletion of the 22q11.2 locus containing most proteins expressed in mitochondria [19] show increased cytoplasmic 8-oxo-2’-deoxyguanosine (suggesting mitochondrial DNA oxidation), reduced number of PV-immunoreactive interneurons, and weakened PNN in anterior cingulate cortex [15]. High 8-oxo-2’-deoxyguanosine alongside with PV neuron defects is a common pathological feature in many animal models relevant to schizophrenia [15]. Following the blockade of NMDARs during early postnatal life, mitochondria in prefrontal PV interneurons show reduced membrane potential and contain high ROS levels [80]. Moreover, mitochondria can act in concert with parvalbumin through homeostatic mechanisms to regulate Ca^2+^ signaling, buffering and sequestration. Thus, parvalbumin modulates mitochondrial volume and dynamics by altering fusion, fission and mitophagy [115]. In PFC of Gclm KO mice, we identified a novel molecular mechanism linking mitochondria and OxS-induced PV neuron impairments. OxS induces upregulation of the microRNA miR-137 in PV neurons, leading to decreased COX6A2, a subunit of cytochrome c oxidase complex IV specific to PV neurons, and to impaired mitophagy with accumulation of damaged mitochondria [116]. Remarkably, the mitochondria-targeted antioxidant, mitoquinone mesylate (MitoQ), rescues this entire pathological process.

### Clinical evidence

Similar alterations of miR-137, COX6A2, and mitophagy markers were identified in plasma of early psychosis patients. Exosomal miR-137 were increased, while COX6A2 and mitophagy markers decreased. Moreover, higher exosomal miR-137 and lower COX6A2 levels were associated with weaker EEG 40-Hz auditory steady-state response. As auditory steady-state response requires proper PV neuron-related networks [116], these suggest that alterations of combined miR-137/COX6A2 plasmatic exosomal levels represent a proxy marker of impairments of cortical PV neuron microcircuits. These findings allowed to stratify early psychosis patients in two subgroups: (a) patients “with mitochondrial dysfunction” characterized by exosomal high miR-137 and low COX6A2, presumably representing PV neuron dysfunction associated with mitochondria and (b) patients “without mitochondrial dysfunction” having miR-137 and COX6A2 levels within the healthy control range. Compared to patients “without mitochondrial dysfunction”, those “with mitochondrial dysfunction” exhibit impaired auditory steady-state response, worse psychopathological status, neuro-cognitive performance and global and social functioning. In this context, it should be noted the robust genetic association between miR-137 polymorphisms and schizophrenia in large-scale GWAS studies [117].

Altogether, these results suggest that exosome-based miR-137 and COX6A2 levels are biomarkers of a PV neuron energy metabolism deficit and gamma oscillation alterations leading to an excitatory/inhibitory imbalance related to various schizophrenia symptoms and functional outcome. This study paves the way for biomarker-guided treatment targeting mitochondrial impairments in a specific subgroup of patients. It also allows monitoring the effect of an intervention relying on both peripheral and central markers. Thus, future stratified clinical trials with mitochondria-targeted antioxidants are warranted. These novel findings highlight a compromised mitochondrial function in PV neurons of schizophrenia patients that may critically act in a feed-forward regulatory loop contributing to their OxS-driven deficits (Fig. 1).

## Reciprocal interactions between NMDA-receptor (NMDAR) hypofunction and redox dysregulation/oxidative stress

Compelling evidence supports the hypothesis of a hypofunction of NMDARs as one mechanism contributing to psychosis and schizophrenia pathology [6, 118, 119]. Genetic risk factors related to NMDARs or associated proteins [20, 120, 121] suggest that hypofunction of NMDARs and mediated signaling pathways could disrupt normal brain maturation, thus contributing to the emergence of schizophrenia. The pathological mechanisms associated with NMDAR dysfunction during early postnatal development have been unveiled by series of preclinical studies pointing to the involvement of OxS [20]. Transient blockade of NMDARs by antagonists during early postnatal life causes at adulthood behavioral phenotypes relevant to schizophrenia [122–124]. Such perinatal functional disruption of NMDARs causes a persistent oxidative state of GSH and prominent OxS in prefrontal PV neurons [80]. This has a long-term impact on PV neurons [125, 126]. But, NAC alleviates both behavioral anomalies [123] and PV neuron impairments [80]. Notably, mice lacking the NADH-oxidase-2, an enzyme that produces superoxide, are resilient to perinatal ketamine-induced PV neurons defects [126]. A genetic model of NMDAR hypofunction relevant to schizophrenia, the D-serine racemase KO mouse which show altered neuronal oscillations [127], also have reduced number of prefrontal PV interneurons together with OxS [15], both of which can be prevented by an early-life NAC treatment (coll. with Joe Coyle, unpublished). Collectively, these indicate that a disruption of NMDAR function during postnatal development affects normal maturation of PV neurons via mechanisms related to OxS [20, 126]. Cortical PV interneurons undergo an early postnatal and activity-dependent switch of the GluN2 subunit composition of NMDARs, with GluN2A becoming more numerous than GluN2B subunits during the time of maturation of these neurons [128, 129]. We have shown that a genetic deletion of GluN2A delays the maturation of prefrontal PV interneurons and PNN, but also reduces the expression of genes coding for enzymes related to GSH and peroxiredoxin systems [130]. Thus, functional deletion of GluN2A renders PV interneurons susceptible to an oxidative insult during their critical period of maturation leading to long-lasting PV neuron/PNN anomalies and reduced high-frequency neuronal synchrony that are prevented by NAC [130]. Likewise, a specific deletion of the obligatory GluN1 subunit of NMDARs in forebrain interneurons, mostly composed of PV interneurons, leads to increased OxS in PV neurons following social isolation. This is associated with reduced expression of genes involved in several antioxidant systems [131]. Indeed, synaptic NMDAR activity boosts intrinsic antioxidant defenses via transcriptional control of thioredoxin/peroxiredoxin [132] and GSH systems, and enhances the synthesis, recycling and utilization of GSH [133]. This suggests that neurons use NMDAR-mediated signaling to adjust the strength of antioxidant defenses accordingly to their activity and metabolic demand, a phenomenon particularly vital for fast-spiking PV neurons. Noteworthy, deletion of GluN1 also causes reduced expression of the transcriptional coactivator PGC1-alpha, which is highly expressed in PV interneurons and is a positive regulator of the expression of genes implicated in mitochondrial function and antioxidant defence [131].

On the other side, oxidative conditions negatively and reversibly modulate NMDAR activity via extracellular redox-sensitive sites located on GluN1-GuN2A receptors [134, 135] and inhibition of CaMKII activity [136]. Notably, GSH deficiency induces NMDAR hypofunction and long-term potentiation impairment [137]. Overall, the reciprocal interactions between NMDAR hypofunction and redox dysregulation/OxS can perpetuate vicious feed-forward mechanisms particularly deleterious for the maturation and function of PV neurons (Fig. 1).

## Reciprocal interactions between neuro-inflammation and redox dysregulation/oxidative stress

OxS and inflammation are reciprocally interconnected [138] and can activate each other. Increased inflammation was reported both in brain and blood of schizophrenia patients [139, 140], originated by complex interaction between genetic [9, 117] and environmental risk factors such as perinatal infections [141] and childhood trauma [142], triggering the release of pro-inflammatory cytokines that in turn promote free radical production. In Gclm KO mice, we have identified a vicious feed-forward process between OxS and neuro-inflammation occurring early during brain development, which underlies the long-lasting effect on PV neuron/PNN integrity [90]. This pathological mechanism involves the following sequential steps: 1) activation of the redox-sensitive metalloproteinase-9 (MMP9) by a redox dysregulation; 2) shedding of the receptor for advanced glycation end-products (RAGE) into a soluble part and an intracellular domain which translocates to the nucleus; 3) activation of the nuclear factor-kB; and 4) secretion of pro-inflammatory cytokines leading to microglia activation and further ROS production which in turn perpetuates OxS-mediated processes from the juvenile stage to adulthood. Blockade of MMP9 activation during the PV neuron maturation period (early peripuberty) prevents RAGE shedding, microglia activation and OxS, and allows normal maturation of PNN and PV neurons. Translation of these findings to early psychosis patients revealed elevated soluble RAGE (sRAGE) in the plasma of patients compared to healthy controls [90], an effect reversed by NAC [143]. In early psychosis patients with high-risk GCLC genotypes, this increased level of circulating sRAGE was associated with low GABA levels in PFC, potentially implying a central inhibitory/excitatory imbalance linked to shedding of RAGE and highlighting the importance of the genetic vulnerability to redox dysregulation [90]. These new findings set a precedent for mechanistic biomarkers needed for early intervention in psychosis and suggest that MMP9/RAGE pathway modulation may also lead to promising drug targets. Summing up, these results support the concept of reciprocal vicious feed-forward interaction processes between microglia activation and OxS leading to PV neuron impairments (Fig. 1).

## Oxidative stress, dopamine dysregulation and PV neuron impairment in the ventral hippocampus

Research using ^18^F-dopa Positron Emission Tomography (PET) indicates that clinical high-risk subjects who convert to psychosis show elevated presynaptic dopamine function in the striatum [144] at baseline, and a progressive increase in striatal dopamine function as they transition [145]. This increased dopaminergic neurotransmission would result in un-sequestered dopamine that can be neurotoxic through its metabolism to form ROS such as hydrogen peroxide and quinones [146, 147]. The ensuing OxS has been implicated in damage to neuronal processes in vitro [148], consistent with the reduction of dendritic spines observed in schizophrenia [149, 150].

Preclinical studies (in MAM rat model or via selective manipulation of PV neurons) have highlighted that PV neuron impairment in ventral hippocampus/subiculum or thalamus reticular nucleus result in overactive ventral-subiculum leading to an increased number of active dopaminergic neurons in the ventral-tegmental-area, which in turn drive elevated dopamine neurotransmission in the mesolimbic system [67, 151–153] (Fig. 1). Indeed, as evidenced by the pioneer works of Grace and collaborators, an increased ventral hippocampal activity causes the nucleus accumbens to strongly inhibit the ventral pallidum, which in turn increases the number of spontaneously active ventral-tegmental-area dopamine neurons [151, 154]. Interestingly in MAM rats, OxS-induced impairments of PV neurons in thalamus reticular nucleus [56] lead to the disinhibition of the multisynaptic excitatory pathway “infralimbic-cortex/reuniens/ventral-subiculum”, contributing to the ventral-subiculum hyperactivity and the consequent dopamine hyperactivity [153]. NAC treatment prevented the PV deficits in thalamus reticular nucleus and dopamine dysfunction, suggesting that early antioxidant treatment might contribute to dopamine normalization in schizophrenia.

## Macrocircuit dysfunction and redox dysregulation in relation to disrupted myelination and white matter integrity diffusion properties

In addition to PV neurons, oligodendrocytes are highly sensitive to altered redox state [155]. Brain diffusion MRI studies show spatially widespread white-matter (WM) abnormalities [156, 157] whose severity increases as the disease progresses [158]. However, from a topological point of view, WM alterations tend to concentrate within fibers interconnecting hub regions and comprising the rich club [159]. The rich club is an organizational property of the brain network that results from a propensity of central brain regions or “hubs” to be more likely interconnected among each other than expected by chance, providing faster routes of transfer and efficient integration of information between remote and separated brain regions [160]. Interestingly, these hub regions which process large amounts of information have high metabolic requirements, are characterized by the co-expression of genes regulating oxidative metabolism, and might be particularly sensitive to OxS [161]. WM abnormalities typically result from dystrophic alterations of oligodendrocytes at the ultrastructural, genetic, epigenetic and molecular levels [162–164]. Similarly to PV neurons, oligodendrocytes have a high metabolism to build and maintain the myelin sheets around the axons [165] and express elevated antioxidant enzymes (catalase and GPx) to prevent lipid peroxidation [166]. In addition, these glial cells contain elevated levels of iron needed as co-factor for many enzymes implicated in myelin synthesis. These glial cells are therefore particularly vulnerable to OxS. Environmental risk factors for schizophrenia generating OxS affect maturation and maintenance of oligodendrocyte integrity [167]. Thus, a dysregulated homeostasis between energy metabolism and antioxidant machinery may have deleterious effects on the maturation, structural and functional integrity of WM [155]. Of note, oligodendrocytes of schizophrenia patients have reduced volume and number of mitochondria [164].

From a developmental perspective, cellular redox state plays a vital role in maintaining the balance between proliferation and differentiation of oligodendrocyte precursor cells in the developing CNS, with a more oxidized state associated with their differentiation whereas a reduced state promotes their proliferation [168–170]. Thus, a redox dysregulation resulting from a GSH deficit modulates the switch from cell proliferation to early differentiation via alteration of the Fyn kinase pathway and impairs late differentiation [168, 171]. ROS can also inhibit the mTOR-P70S6K signaling cascade leading to decreased protein synthesis for proliferation and differentiation [172]. Of note, post-mortem analysis suggests impaired differentiation of oligodendrocyte precursors in schizophrenia [173]. Likewise, we found that mice with a GSH deficit (Gclm KO) exhibit reduced numbers of mature oligodendrocytes and myelin markers, suggesting that dysregulation of Fyn kinase pathway may underlie these anomalies [168]. In this context, the regulation of Fyn mRNA and protein expression is impaired in fibroblasts from schizophrenia patients with genetic risk for GSH deficit [168]. The importance of GSH is further supported by the positive correlation between patients GSH levels in PFC and structural WM diffusion properties in the cingulum bundle [168]. Other evidence linking GSH deficit to WM stems from a 14 T diffusion MRI longitudinal study on Gclm KO mice. This revealed reduced fractional anisotropy within the fornix/fimbria accompanied by a slower conduction velocity along nerve fibers [174]. Similarly WM diffusion properties were decreased in fornix of early psychosis patients, in correlation with a smaller hippocampus volume and elevated blood oxidative status marker [175].

As for PV neurons, complex reciprocal interactions between redox dysregulation/OxS, mitochondria, neuroinflammation, and NMDAR function may generate vicious effects for oligodendrocytes (Fig. 1). The differentiation and integrity of oligodendrocytes require a coordinated regulation of metabolic needs and redox balance to prevent the deleterious effect of OxS, also triggered by neuroinflammation. Oligodendrocytes are vulnerable to early-life neuroinflammation [176, 177] leading to impaired myelination [167, 172, 178, 179]. As immune targets and regulators, oligodendrocytes are engaged in multiple cross-talks with microglia that include responses to stress which can lead to myelin damages, but also mechanisms of repair [180].

NMDAR dysfunction may also lead to alterations of WM diffusion properties. Indeed, stimulation of NMDARs, expressed in immature and mature oligodendrocytes, promote the maturation of these cells and myelination around axons [181–183], up-regulates their energy metabolism, increases mitochondria motility within myelin sheath, and glycolytic support to the axons [184, 185]. Thus, a dysfunction of NMDARs on oligodendrocytes, which remains speculative in schizophrenia, affects myelination and proper regulation of the energy coupling between oligodendrocytes and axons. This could be especially detrimental for fast-spiking neurons such as PV interneurons whose axons are strongly myelinated [186, 187].

## A focus on redox dysregulation in relation to childhood trauma

Evidence supporting the interplay of genetic and environmental factors in relation to liability for schizophrenia stems from human and animal studies. Traumatic experiences occurring during the critical time of childhood and adolescence favors the development of psychiatric disorders associated with psychosis and cognitive impairments [188, 189]. Supported by studies on animal models, some symptoms and cognitive deficits may be directly associated to the deleterious impact of these environmental stresses on PV neurons and oligodendrocytes through the action of OxS. In rats, stress during adolescence, but not adulthood, leads to long-term hyperactivity of the dopaminergic system that is relevant to positive symptoms [190] and is concomitant to deficits of hippocampal PV interneuron-networks [101]. Our data suggests this is due to a high vulnerability of PV interneurons to OxS during childhood and adolescence as opposed to adulthood [81]. Early-life stress increases OxS in prefrontal and hippocampal PV interneurons [191]. Goodwill et al. (2018) [192] also show that early-life stress causes long-term decreased PV expression and density of PV neurons in orbitofrontal cortex which result in impaired rule-reversal learning. Early-life induced persistent decrease of PV expression in PFC is due to HDAC1-dependent epigenetic mechanisms [94]. Likewise, prepubertal stress exacerbates the effects of a previous maternal immune challenge leading to significant OxS, deficits in PV neurons and PNN in PFC [15].

Prenatal stress as well as trauma during childhood and adolescence also affect WM properties [193–195]. However, the impact of stress during these developmental periods on oligodendrocytes is not fully documented. In mice, social isolation through childhood and adolescence, known to affect PV neurons through OxS [83], has also persistent effect on oligodendrocyte morphology and density, and causes reduced expression of myelin-associated proteins and myelin thickness [196]. Altogether, this suggests that redox dysregulation/OxS play a role on the impact of childhood traumatic experiences in patients suffering from schizophrenia.

With this in mind, we assessed a cohort of early psychosis patients in which some had been exposed to severe childhood trauma (sexual and physical abuse). Interestingly, exposure to trauma (particularly when exposed before age of 12 years) is associated with severe positive, negative and depressive symptoms, bad functional and social outcome [197]. This was in contrast to early psychosis patients exposed to trauma after 12 years of age who mostly suffered from negative symptoms and had a similar functional outcome to the non-trauma-exposed early psychosis patients [197, 198]. Among early psychosis patients exposed to childhood trauma, we recently identified two separate groups. One group with high peripheral oxidation status (high GPx activity) displayed smaller hippocampal volumes and more severe symptoms, while the other group with lower oxidation status (low GPx activity) showed better cognition and regulation of GSH- and thioredoxin/peroxiredoxin- systems [199]. These results suggest that maintained regulation of various antioxidant systems allows compensatory mechanisms for mitigating long-term neuroanatomical and clinical impacts. The redox marker profile may thus be useful to define treatment strategies at early stages of psychosis.

## From bench to bedside: a focus on clinical trials with N-Acetyl-Cysteine (NAC)

New treatment strategies are increasingly interested in antioxidant compounds such as NAC [200–202]. NAC is reported to have beneficial effects on negative symptoms [203–205] and cognition [205–207] in patients with chronic schizophrenia and with first psychotic episode [208, 209]. It also improved EEG mismatch negativity [210] and local synchronization [211]. In a recent randomized controlled trial on early psychosis patients, we observed that a 6-month NAC add-on treatment significantly increased the levels of GSH in PFC, suggesting a good drug-target engagement [209]. NAC improved neurocognitive-processing speed in correlation with negative symptoms. Interestingly, our study found that NAC could also improve positive symptoms but only in early psychosis patients exhibiting a high blood oxidative status [209]. We have also shown for the first time that NAC administration to early psychosis patients improved WM diffusion properties in fornix. This improvement was correlated with brain GSH increase [212]. In addition, NAC ameliorated low-level auditory processing [213] and resting-state functional connectivity within the cingulum bundle [214]. A single dose monotherapy with NAC reduced medial frontal resting-state functional connectivity [215]. Taken together, these findings open the gateway to biomarker-guided therapy. Nonetheless, further longitudinal studies of antioxidant treatment in larger cohorts of biomarker selected patients, controlled by target engagement, are required.

## Potential interventions for breaking the vicious circles of oxidative stress

**Fig. 2:**
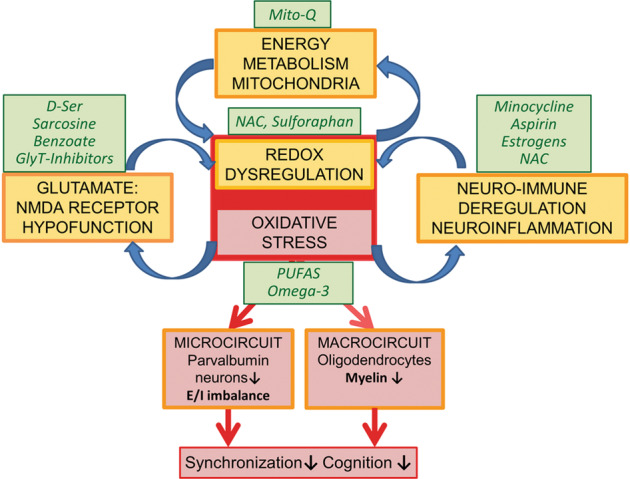
Potential points of action of various molecules capable of interrupting the diverse vicious circles. **a** Treatments with compounds such as NAC, sulforaphane or omega-3 type polyunsaturated fatty acids (PUFAS)—which possess both anti-oxidative and anti-inflammatory properties with mild side-effect profiles [202, 218–221] are known to exert their effects through the redox system thus representing good candidates for preventive interventions targeting individuals at high-risk for schizophrenia. Sulforaphane works through the NRF2 system to trigger the anti-oxidant defence system [222]. **b** MitoQ and other mitochondria-targeted antioxidants could support compromised energy metabolism and mitochondria function [116]. **c** For NMDAR hypofunction, several strategies using compounds (e.g., D-serine, sarcosine, glycine transporter inhibitor and benzoate) that modulate NMDAR activity have been tried in schizophrenia patients with mixed success [217, 223–229]. **d** In terms of neuro-inflammation, estrogens, minocycline and NAC showed efficacy, with greater beneficial results on symptom severity in first-episode psychosis patients or during early-phase of schizophrenia [216].

Interventions/drugs that aim at breaking the different vicious circles causing persistent OxS represent promising strategies to reduce the deleterious effects on PV neurons and myelin-forming oligodendrocytes [14], and therefore mitigate the emergence or severity of the disorder. In this context, one should consider compounds with both anti-oxidative and anti-inflammatory properties (e.g. NAC, sulforaphane, omega-3 polyunsaturated fatty acids) [216], molecules targeting specifically mitochondria (e.g. MitoQ), and positive modulators of NMDAR-mediated signaling (e.g. D-serine, sarcosine, benzoate, glycine transporter inhibitors) [217]. The efficacy of these different compound categories may however differ from patients to patients, according to the timing of initiation during neurodevelopment of the vicious feedforward processes that are primarily triggered and the disease stages (prodrome, first episode or chronic). Biomarker-based approaches, targeting validated mechanisms, will be essential to identify individuals more likely to respond to a specific drug in future clinical trials (Fig. 2).

A question remains: could interventions that manipulate OxS mechanisms be applied for patients exposed to childhood trauma in real world clinic setting? In Gclm KO mice, we recently showed that the sequential combination of NAC treatment and environmental enrichment applied during the juvenile and adolescent periods respectively normalizes the integrity and function of PV neuron/PNN networks induced by an additional oxidative insult during childhood, that mimics childhood adverse events that would induce OxS [143]. NAC, via inhibition of OxS-induced MMP9/RAGE pathway, interrupts the deleterious feedforward mechanism that maintains persisting high OxS levels and neuroinflammation, allowing PVI/PNN maturation (see chapter 5). A subsequent environmental enrichment during adolescence promotes the final maturation of PV neurons, providing a long-term neuroprotection to PV neuron/PNN networks. Translating to early psychosis patients, a 6-month NAC treatment reduces plasma sRAGE in association with increased prefrontal GABA, improvement of working memory, processing speed and positive symptoms, suggesting similar neuroprotective mechanisms [143]. Thus, by analogy, in real world clinic, patients exposed to early-life insults may benefit from a biomarker (sRAGE/MMP9)-guided antioxidant treatment combined with the equivalent of “environmental enrichment”, which could include physical training, nutrition, social activities and psychotherapy.

## Conclusion

Collectively the above data support the view that the various genetic and environmental factors impinging on one or more pathological mechanisms involved in schizophrenia, namely anomalies in mitochondria and energy metabolism, NMDAR hypofunction, neuroinflammation will ultimately impair redox regulation leading to OxS and its deleterious consequence on PV neurons and oligodendrocytes. As shown for PV neurons, a redox dysregulation can in turn further promote NMDAR hypofunction, mitochondrial impairment, and neuroinflammation in feed-forward vicious circles that perpetuate the persistence of OxS and long-term impact on these highly metabolic cells. Eventually, a primary dysfunction of NMDARs, or neuroinflammation, or mitochondrial dysfunction, or impaired regulation of antioxidant systems could involve in turn the other vicious circles, all converging to common deleterious impacts on PV neurons and oligodendrocytes during neurodevelopment (Fig. 1). In addition to psychotic disorders and schizophrenia, the proposed mechanisms may also be applied to other psychiatric diseases including autism and bipolar disorder.

By adopting the reverse translation of validated circuitry- relevant human endpoints approach [2], we provide convincing proof-of-concept for targeting OxS through antioxidant-based strategies in individuals with schizophrenia and underscore the importance of “breaking” the various vicious circles associated with OxS as means to prevent the propagation of processes that may precede the onset of disease. To improve early detection and increase the signal-to-noise ratio for adjunctive trials of antioxidants, anti-inflammatory and NMDAR modulator drugs, the above presented processes allow to identify mechanism-based biomarkers guiding stratification of homogenous patients groups and target engagement required for successful clinical trials, paving the way towards precision medicine in psychiatry [90, 116, 143, 209, 212]. Presently, it is not easy to interfere with the genetic component of the disease nor fully prevent the impact of environmental factors. Thus, acting early during development on the vicious circles leading to lasting OxS might be a rewarding strategy to reduce its consequences on key functions of micro- and macro-circuits impairments and their clinical manifestations.
